# Novel Bioequivalent Tablet of Solifenacin Succinate Prepared Using Direct Compression Technique for Improved Chemical Stability

**DOI:** 10.3390/pharmaceutics15061723

**Published:** 2023-06-14

**Authors:** Do Hwan Kim, Myoung Jin Ho, Chan Kyu Jeong, Myung Joo Kang

**Affiliations:** 1 College of Pharmacy, Dankook University, 119 Dandae-ro, Dongnam-gu, Cheonan-si 31116, Chungcheongnam-do, Republic of Korea; porimikdh@daum.net (D.H.K.); butable@gmail.com (M.J.H.); 2 Shin Poong Pharm. Co., Ltd., 1203-ho Daerung Techno Town 15, 401, Simin-daero, Dongan-gu, Anyang-si 14057, Gyeonggi-do, Republic of Korea; jazzjeong@naver.com

**Keywords:** solifenacin succinate, direct compression, solid dosage form, storage stability, pharmacokinetics, bioequivalence

## Abstract

We designed a bioequivalent tablet form of solifenacin succinate (SOL) with an improved storage stability using a direct compression (DC) technique. An optimal direct compressed tablet (DCT) containing an active substance (10 mg), lactose monohydrate, and silicified microcrystalline cellulose as diluents, crospovidone as a disintegrant, and hydrophilic fumed silica as an anti-coning agent was constructed by evaluating the drug content uniformity, mechanical properties, and in vitro dissolution. The physicochemical and mechanical properties of the DCT were as follows: drug content 100.1 ± 0.7%, disintegration time of 6.7 min, over 95% release within 30 min in dissolution media (pH 1.2, 4.0, 6.8, and distilled water), hardness > 107.8 N, and friability ~0.11%. The SOL-loaded tablet fabricated via DC showed an improved stability at 40 °C and RH 75%, exhibiting markedly reduced degradation products compared to those fabricated using ethanol or water-based wet granulation or a marketed product (Vesicare^®^, Astellas Pharma). Moreover, in a bioequivalence study in healthy subjects (*n* = 24), the optimized DCT offered a pharmacokinetic profile comparable to that of the marketed product, with no statistical differences in the pharmacokinetic parameters. The 90% CIs for the geometric mean ratios of the test to the reference formulation for the area under the curve and the maximum drug concentration in plasma were 0.98–1.05 and 0.98–1.07, respectively, and satisfied the FDA regulatory criteria for bioequivalence. Thus, we conclude that DCT is a beneficial oral dosage form of SOL with an improved chemical stability.

## 1. Introduction

Solifenacin succinate (SOL), a competitive muscarinic antagonist with high selectivity for the M3 receptor in the urinary bladder, is prescribed to treat overactive adult bladder symptoms [1,2,3]. After a 12-week phase III study, patients who orally received 5 or 10 mg of SOL showed marked reductions in the number of voids, incontinence episodes, and urgency episodes per 24 h [4]. Although the muscarinic antagonist is lipophilic (log*p* value of 1.69, at pH 7.0), its succinate salt is sparingly soluble (10 mg/mL, pH 7.2) in aqueous media [5]. The plasma SOL levels peaked 3–8 h after oral administration (5 or 10 mg tablets). The drug has a high bioavailability of over 88% in healthy participants [6,7]. Currently, the drug is commercially formulated as immediate-release (IR) tablets (Vesicare^®^, Astellas Pharma Europe, B.V., Leiden, The Netherlands). It was fabricated via a wet granulation process employing maize starch and lactose monohydrate as diluents, hypromellose as a binder, magnesium stearate and macrogol as lubricants, and talc and titanium dioxide as anti-caking agents [8]. During wet granulation using organic solvents or distilled water, amorphous active compounds can be produced using pharmaceutical excipients [9,10,11]. The crystalline form of SOL is chemically stable under harsh conditions; however, the amorphous form of SOL is extremely unstable and forms a substantial amount of degradation products under high temperature and high humidity conditions [12]. The amorphous form of SOL is easily oxidized, producing several degradation products via N-oxide at the quinuclidine ring or benzylic radical formation [13]. Therefore, sophisticated formulation studies are essential for improving the chemical stability of muscarinic antagonists.

The direct compression (DC) method is the first choice for preparing tablets [14]. The production process includes blending the active substances with pharmaceutical excipients and lubricants, followed by tabletization, with no additional processing steps. It offers the following several advantages: (i) it is more economical than the wet granulation process because it requires fewer unit operations; (ii) it is a better choice for moisture- and heat-sensitive APIs because the wetting and drying steps are eliminated; and (iii) drug crystalline conversion during the wet granulation and drying processes can be avoided, providing a stable dissolution profile [15,16,17]. However, a crucial selection of pharmaceutical excipients is required compared to those used for granulation to ensure the appropriate flowability and compressibility of drug powders. Moreover, problems may arise when low amounts of active compounds need to be incorporated into tablets, because it is challenging to accurately blend a small amount of active ingredients in a large amount of excipient to achieve the desired uniformity and homogeneity. Despite the advantages of the DC method, to the best of our knowledge, no SOL-loaded tablet dosage forms have been fabricated using this technique.

The principal goal of this study is to design a new tablet dosage form of SOL using the DC technique to simultaneously ensure improved chemical stability and a bioequivalent pharmacokinetic profile compared to those of the commercial product. The tablet composition was established by evaluating the drug content uniformity, mechanical properties, and in vitro dissolution. The chemical stability of the direct compressed tablet (DCT) in terms of drug content and degradation substances was evaluated and compared with that of the tablets prepared via wet granulation using distilled water (DWT) or ethanol (ETT), and with that of the marketed product. Furthermore, the bioequivalence of the DCT with a commercial SOL tablet fabricated using wet granulation was evaluated in healthy adult male participants.

## 2. Materials and Methods

### 2.1. Materials

Powdered SOL was purchased from MSN Laboratories Ltd. (Hyderabad, India). The median diameter (D_50_) of the drug powder was approximately 80 μm, with D_10_ (10% of the smaller particles contained) and D_90_ (90% of the total smaller particles in the sample) values of 30 and 150 μm, respectively. Lactose monohydrate 200 mesh, Supertab 30GR (agglomerated lactose monohydrate), and Flowlac100 (spherical lactose monohydrate) were obtained from DFE Pharma (Veghel, The Netherlands), DFE Pharma (Norten Hardenberg, Germany), and Molkerei Meggle Wasserburg (Wasserburg, Germany), respectively. Several grades of microcrystalline celluloses (MCCs) such as Prosolv SMCC50 and 90 (silicified MCCs), Vivapur pH102, and Vivapur-12 were obtained from JRS Pharma (Weissenborn, Germany). Crospovidone (Kollidon CL) and vinylpyrrolidone–vinyl acetate copolymer (Kollidon VA64) were obtained from BASF (Ludvigshafen, Germany). Magnesium stearate and sodium stearyl fumarate were obtained from Faci (Jurong Island, Singapore) and JRS Pharma (Polanco, Spain). Hydrophilic fumed silicone dioxide (Aerosil 200) and pink film-coating material (Opadry^®^ 03B640016, mainly composed of hypromellose 2901, titanium dioxide, and iron oxide) were provided by Evonik (Rheinfelden, Germany) and Colorcon (Shanghai, China), respectively. Hypromellose 2910 (6 cps), cornstarch, croscarmellose sodium, and sodium starch glycolate were provided by Lotte Fine Chemicals (Incheon, Republic of Korea), Duksan Pure Chemicals (Ansan-si, Republic of Korea), JRS Pharma (Pirna, Germany), and Yung Zip Chemicals (Talchung, Taiwan). Pharmaceutical standards for the potent degradation products of SOL, such as solifenacin N-oxide (purity ≥ 98%), YM217880 ((+)-(R)-quinuclidin-3-yl [2-(2-benzoylphenyl)ethyl]carbamate) (≥98%), isoquinoline ((1S-1-Phenyl-1,2,3,4-tetrahydro-2-isoquinoline) (≥98%), and isoquinoline ester ((1S-ethyl-1-Phenyl-1,2,3,4-tetrahydro-2-isoquinoline carboxylate) (≥98%) were obtained from MSN Laboratories, Ltd. (Hyderabad, India). All organic solvents, including acetonitrile (ACN), methanol, and methyl tert-butyl ether, were of high-pressure liquid chromatography (HPLC) grade and were used without further purification.

### 2.2. Preparation of SOL-Loaded Tablets Using DC Technique

The DCTs (DCT1-DCT11) listed in Table 1 and Table 2 were fabricated on a 1000 T scale using the DC method. The active substances, diluents (corn starch, Vivapur-12, MCC pH102, Prosolv SMCC50, or Prosolv SMCC90), binder (hypromellose 2910 or Kollidon VA64), disintegrants (croscarmellose sodium, sodium starch glycolate, or Kollidon CL), and anti-coning agent (Aerosil 200) were accurately weighed, mixed using bag mixing, and sieved through a 40 mesh twice. Thereafter, diluents (lactose monohydrate 200 mesh, Flowlac100, or Supertab 30GR) were added to the mixture, which was then lubricated with the lubricant (magnesium stearate or sodium stearyl fumarate). The powder mixtures were compressed using a single-punch tablet press (EKO-D, Korsh, Berlin, Germany) (DCT1, DWT, and ETT) and a rotary tablet press (KT06SS, Keumsung, Gyeonggi-do, Republic of Korea) (DCT2-DCT11). In the single-punch tableting process, the mixture was filled into a die using a feeder and manually compressed. The rotary tablet press was equipped with six round convex Euro D punches with a 7.5 mm diameter (YoungChang punch Co., Ltd., Yongin, Republic of Korea). The fill depth was adjusted to obtain 150 mg tablets. The turret speed (or tableting speed) was varied between 10 rpm and 30 rpm. The hardness of the tablets was controlled by adjusting the height of the upper punch. Opadry 03B640016 was dissolved in water at a concentration of 10% *w*/*v* and used as the coating solution. The coating solution was sprayed onto the SOL-loaded tablet using Thai coater (PMS, Bangkok, Thailand), with a fan speed of 80 rpm, spray speed of 5.1 mL/min, inlet temperature of 105 °C, and product temperature of 40 °C.

### 2.3. Preparation of SOL-Loaded Tablets Using Wet Granulation Method

Additional sol-loaded tablets were prepared using the water- and ethanol-based wet granulation methods (DWT and ETT, respectively). For both DWT and ETT, the drug powder was mixed with cornstarch as a diluent and sieved through a 40-mesh sieve. Lactose monohydrate (200 mesh) was added to the drug and cornstarch mixture and mixed for 5 min using a high-speed mixer (Diosna P1-6, DIOSNA Dierks & Söhne GmbH, Osnabrück, Germany) at an impeller speed of 350 rpm and a chopper speed of 2200 rpm. To prepare the DWT, hypromellose 2910 (6 cps) was added to purified water and stirred using an overhead stirrer until completely dissolved [18]. For the ETT, hypromellose 2910 (6 cps) was added to 95% ethanol and fully dissolved using an overhead stirrer. The binder solution was added dropwise over 30 s and granulated for 1 min using a high-speed mixer. The granules were desiccated at 60 °C using a tray dryer, until the loss of drying (LOD) value reached below 3 *w*/*w*%. The dried granules were passed through a 20-mesh sieve using an oscillating granulator (Erweka^®^ FGS, Erweka GmbH, Langen, Germany). The granules were lubricated with 40-meshed magnesium stearate using a V-mixer for 5 min at 20 rpm. Subsequently, the granules were compressed using a single-tablet press machine in the same manner as described in Section 2.2.

### 2.4. Drug Content and Degradation Product Analysis 

The drug content and degradation products of the SOL-loaded tablets were determined via HPLC [19]. To determine the drug content, 10 tablets were placed in a mixture of ACN and DW (35:65, *v*/*v*, 100 mL), followed by bath sonication for 1 h to disintegrate and extract the active substance from the solid dosage form. The samples were ultracentrifuged at 13,000 rpm for 10 min, and the drug concentration in the supernatant was analyzed using HPLC. The Waters HPLC system consisted of an autosampler (Model 717 plus), pump (Model 515 pump), UV-VIS detector (Model 486), and Empower^®^ software. The mixture of phosphate buffer (0.05 M K_2_HPO_4_, pH 6.0 adjusted with phosphoric acid) and ACN (65:35, *v*/*v*) was used as the mobile phase, and was supplied into C_18_ column (Xbridge^™^, 4.6 × 150 mm, 5 μm) at a flow rate of 1.0 mL/min. The injection volume was 10 μL. The column eluent was detected at a wavelength of 210 nm, with a retention time of 6.9 min. The calibration curve was linear between 1.0 and 100 μg/mL, with an *r*^2^ value of 1.0. 

The SOL degradation products in the tablets were analyzed separately using HPLC. Ten tablets were immersed in a mixture of methanol and ACN (4:3, *v*/*v*; 100 mL), and the drug was dissolved in the solvent via bath sonication for 1 h. After centrifugation, the supernatant was diluted via the mobile phase and injected into a Shimadzu HPLC system composed of a pump (Model 515 pump), an autosampler (Model 717 plus), and a UV–VIS detector (Model 486). The mobile phase consisted of a phosphate buffer (dipotassium hydrogen phosphate 3.4 g/L, trifluoroacetic acid 1 mL/L, pH 7.5) and methanol (35:65 *v*/*v*). The mobile phase was run on an analytical column at a flow rate of 1 mL/min. The column temperature and injection volume were set to 35 °C and 20 L, respectively. The eluent was monitored at 210 nm for 60 min. The level of related substances (%) was calculated as the peak area of individually related substances compared to the corresponding peak area of the active substance.

### 2.5. Disintegration Time of SOL-Loaded Tablets

The disintegration rates of the different SOL-loaded tablets were assessed by following the USP pharmacopeial method (KDIT-200; Kukje Eng, Goyang, Republic of Korea) [20,21]. The disintegration apparatus consisted of a basket-rack assembly, a 1000 mL beaker, a thermostatic arrangement for heating the fluid, and a mechanical device to escalate and lower the basket in the immersion fluid at a constant frequency. Six tablets from each series were immersed in 900 mL of purified water maintained at 37 ± 0.5 °C, and the disintegration time of the formulas were determined visually.

### 2.6. Mechanical Properties of SOL-Loaded Tablets

The mechanical properties, including hardness and friability (%), of the SOL-loaded tablets were assessed using pharmacopeial methods [22]. Hardness (Netwon, N) of the tablets was determined using a hardness tester (Erweka TBH201, *n* = 10 per formula). The friability of SOL-loaded tablets was measured using a friability tester (KTF-100, Kukje Eng, Gyeonggi-do, Republic of Korea). After weighing, tablets were placed in a drum and rotated 100 times. Any loose dust from the tablets was removed, and the weight of the tablets was measured. The decrease in weight (%) relative to the initial weight of the SOL-loaded tablets was calculated.

### 2.7. In Vitro Dissolution Profile of SOL-Loaded Tablets

The in vitro dissolution pattern of SOL from the DCTs was evaluated using the USP Paddle 2 method (VK750D, Varian, Huntington Beach, CA, USA) [23]. Simulated gastric fluid (pH 1.2), sodium acetate buffer (pH 4.0), simulated intestinal fluid (pH 6.8), and distilled water were used as the dissolution media. Prior to dissolution test, about 900 mL of the medium was added to the vessel, and the temperature of the dissolution media was kept at 37 °C. Subsequently, tablets containing 10 mg of SOL were placed in the vessel and stirred at a paddle speed of 50 rpm. At predetermined times, 10 mL aliquots were withdrawn using a syringe, and the same amount of pre-warmed fresh media was used for replenishment. The sample was then syringe-filtered (PVDF, 0.45 μm) to remove insoluble pharmaceutical excipients or drug particles. The filtrate was diluted with ACN and subjected to HPLC as previously described.

### 2.8. Physicochemical Stability of SOL Tablets under Accelerated Condition

The chemical stability of the SOL-loaded tablets was evaluated under accelerated storage conditions (40 °C/75% RH). DCT1, DWT, and ETT were packaged in polyethylene (PE) bottles containing silica gel and stored in a storage chamber (VP1300, Votsch, Balingen, Germany) for four weeks. For optimized DCT (DCT11), tablets were packaged using alu-alu blister film (Bilcare, Changi, Singapore) and stored in an accelerated chamber (40 °C/75% RH) for 8 weeks. The drug content and degradation products were analyzed via HPLC as described above. The in vitro dissolution profile of the optimized DC tablet after six months of storage under accelerated conditions was also evaluated. 

### 2.9. Bioequivalence Study in Healthy Volunteers

#### 2.9.1. Volunteers and Protocols

The bioequivalence of the optimized DC tablet (DCT11) and the commercial product (Vesicare^®^) was evaluated in healthy Korean male volunteers after obtaining approval from the IRB (Hanaro Medical Foundation, Hanaro Hospital, Seoul, Republic of Korea). All participants participated voluntarily after the recruitment announcement and were selected based on their medical history, blood pathology, blood chemistry, and urine test results. All participants signed a written informed consent form after the purpose, methods, and probable adverse drug reactions of the study were explained to them in accordance with FDA guidelines.

This bioequivalence study was conducted using a randomized, single-dose, two-period crossover design. A total of 32 volunteers were randomly divided into two groups. All participants consumed the same dinner on the day before drug administration, followed by restrictions on exercise, eating, smoking, and consumption of xanthine-containing beverages. Volunteers were fasted for 10 h before and 4 h after drug administration to diminish the effect of food on the pharmacokinetic profile. Volunteers in the two groups were orally administered reference or test tablets containing 150 mL of water at 8 am. Participants were not allowed to drink water for 1 h before or after drug administration. At predetermined time points (0, 1, 2, 3, 4, 5, 6, 8, 12, 24, 48, 96, 144, and 168 h), blood samples (approximately 8 mL) were collected in Vacutainers containing sodium heparin. To prevent blood from clotting, 1 mL of heparinized normal saline was added to the catheter. Blood samples were centrifuged at 3000 rpm for 10 min, and the obtained plasma samples were transferred to Eppendorf tubes and stored at −70 °C until LC/MS-MS analysis. After a washout period of 4 weeks, crossover drugs were administered, and the experiment was conducted using the same protocol as previously described.

#### 2.9.2. LC/MS-MS Analysis of SOL in Plasma 

Plasma SOL levels were analyzed using a validated chromatography–tandem mass spectrometer (LC-MS/MS) comprising a Shiseido nanospace SI-2^®^ chromatography system (Shiseido, Japan) and a Quantum Ultra^®^ mass spectrometer (Thermo, Waltham, MA, USA) [24,25]. As internal standard (IS) for analysis, 10 μL of SOL-d5 (50 ng/mL in 50% methanol) was added to 50 μL of the thawed plasma samples. Thereafter, 1.2 mL of methyl tert-butyl ether was added to the sample and vortexed for 20 min to extract the drug and IS from the plasma. After centrifugation at 12,000 rpm for 5 min, the supernatant was collected and evaporated using nitrogen evaporator (MG-2100, Eyela, Japan) at 50 °C. The samples were then reconstituted with 100 μL of ACN:10 mM ammonium formate/formic acid (50:50:0.1, *v*/*v*/*v*) solution. Afterward, 2 µL of the reconstituted solution was injected onto the liquid chromatography system (Shiseido nanospace SI-2^®^) equipped with C_18_ column (75 mm × 2 mm, 3 μm particle size). The mobile phase consisted of ACN, 10 mM ammonium formate, and formic acid (50:50:0.1, *v*/*v*/*v*). The flow rate of the mobile phase under isocratic conditions was kept at 0.2 mL/min with an analysis time of 3.2 min. The column oven and autosampler temperatures were set at 40 and 10 °C, respectively. 

The analytes were detected using a Quantum Ultra quadrupole mass spectrometer (Thermo, USA) in the positive electrospray ionization (ESI) mode. The MS/MS system was operated at unit resolution in the single reaction monitoring mode, with the precursor ion *m/z* > the product ion *m/z* at 363.3→110.1 and 368.3→110.1 for SOL and IS, respectively. The compound-dependent parameters for SOL and IS were declustering potentials of 86 and 94 V, collision energies of 26 and 27 V, and entrance potentials (V) of 10 and 10 V, respectively. The following MS parameters were used for detection: capillary temperature of 380 °C, spray voltage of 4500 V, nebulizer gas of 2 L/min, drying gas of 10 L/min, and heating gas of 10 L/min. The retention time of SOL and SOL-d5 was approximately 1.68 and 1.67 min, respectively. Data acquisition and quantification were performed using the Xcalibur 2.0.7 (Thermo). This chromatographic method was validated to evaluate its specificity, linearity, precision, and accuracy.

#### 2.9.3. Pharmacokinetic and Statistical Analysis

The pharmacokinetic parameters of SOL following oral administration of SOL-loaded tablets were determined using a non-compartmental model with the BA Calc 2007 program (version 1.0.0; MFDS, Seoul, Republic of Korea). The maximum plasma concentration of SOL (C_max_) and time required to reach C_max_ (T_max_) were determined using the experimental data. The area under the plasma concentration–time curve (AUC) from 0 to 196 h (AUC_0–196 h_) was calculated using the linear trapezoidal method. The elimination rate constant (k_e_) of SOL from the plasma was determined from the least-squares regression slope of the terminal concentration profile. The elimination half-life (t_1/2_) was calculated by dividing 0.693 by k_e_. The AUC from 0 h to infinity (AUC_0–∞_) was calculated as AUC_(0–196 h)_ + C_196 h_/k_e_, where C_196 h_ is the SOL concentration at the last sampling point (196 h). 

The bioequivalence of the optimized DCT (DCT11) compared with the reference was evaluated by estimating 90% confidence intervals (CIs) of the geometric mean ratios of the test to reference values, which were determined using log-transformed data of AUC_0–196_ and C_max_. The K-BE test 2007 (version 1.1.0; MFDS) was employed as the bioequivalence test statistical program [26]. The regulatory range of the 90% CIs of the geometric mean ratios for bioequivalence was 80.00–125.00%.

## 3. Results and Discussion

### 3.1. Effect of Fabrication Process on Chemical Stability of SOL-Loaded Tablets

In order to evaluate the effect of the fabrication process on the chemical stability of SOL in tablets, three different SOL-loaded tablets (DWT, ETT, and DCT1) with different fabrication processes but identical pharmaceutical excipients were prepared, and their chemical stabilities were evaluated under accelerated conditions (40 °C, RH75%). Lactose monohydrate (200 mesh) and corn starch were used as diluents, and hypromellose 2910 (6 cps) and magnesium stearate were used as binders and lubricants, respectively. These pharmaceutical excipients were selected based on the composition of a commercial product (Vesicare^®^) [27]. The LOD values of the DWT, ETT, and DCT1 were comparable, with the values of 2.78 ± 0.25%, 2.57 ± 0.42%, and 2.63 ± 0.31%, respectively (expressed as mean ± SD, *n* = 3). The LOD value in the tablets was determined using a temperature of 105 °C for 10 min. The data denote that the ethanol and purified water added during the granulation process were effectively removed. On the other hand, despite the sufficient drying process, the LOD values in the three SOL-loaded tablets were determined to be over 2.5 *w*/*w*% because of the moisture content included in the pharmaceutical excipients, especially lactose hydrate (4.5–5.5 *w*/*w*% of water content) and corn starch (over 11 *w*/*w*%) [28]. The DWT, ETT, and DCT1 exhibited appropriate mechanical strengths; the hardness of the tablets ranged between 78.5 and 101.0 N, with <0.15% of friability (Table 1). Regardless of the fabrication process, the three types of tablets disintegrated immediately in distilled water, with a disintegration time of 7.8 min (Table 1). In contrast, the DWT, ETT, and DCT1 exhibited markedly different drug stabilities under an accelerated condition (40 °C, RH75%).

**Figure 1:**
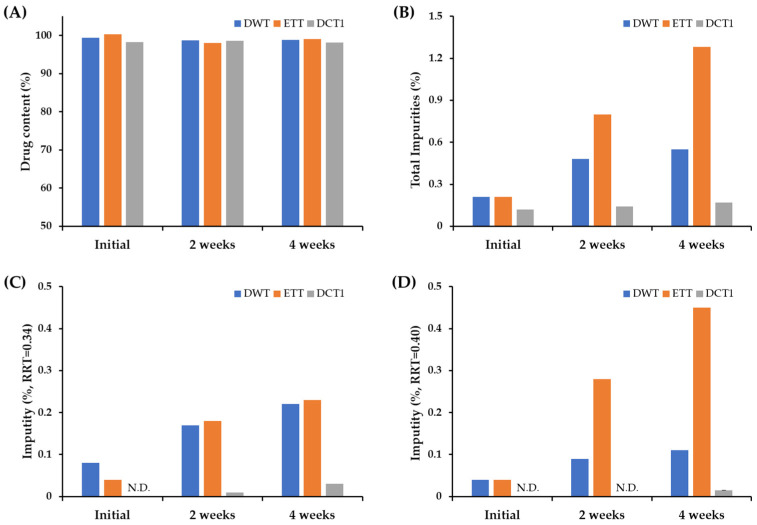
Comparison of the drug content, total impurities, and individual degradation products of the tablets prepared using different fabrication processes. Changes in (**A**) drug content, (**B**) total impurities, individual impurity (%) at (**C**) RRT 0.34 and (**D**) RRT 0.40 in SOL-loaded tablets prepared via wet granulation or DC technique, under accelerated storage condition (40 °C, RH75%). Notes: SOL-loaded tablets (DWT, ETT, and DCT1) were filled into polyethylene (PE) bottle with silica gels and stored in a stability chamber (40 °C, RH75%) for 4 weeks. To determine the drug content (%) and level of degradation products (%) in the tablets, 10 tablets were pretreated and analyzed once. Impurities at RRT 0.34 and 0.40 were identified in solifenacin N-oxide and YM217880, respectively. N.D. = not detected.

Although the drug content of all the formulations remained stable (over 98.1%) for four weeks (Figure 1A), markedly higher amounts of degradation products were observed in the DWT and ETT than in the DCT1 (Figure 1A). In the HPLC analysis, the degradation products of SOL were detected at relative retention times (RRT) of 0.30, 0.34, 0.40, 0.43, and 0.51 min, respectively (Figure S1). In the ETT, the related substances were eluted at RRT values of 0.30, 0.34, 0.37, 0.40, 0.41, 0.44, and 0.51 (Figure S1). In the DWT and ET, the peak areas of RRT 0.34 and RRT 0.40 were quantified to be over 0.1% compared to that of the active substance, whereas those of the other degradation products were less than 0.1%, indicating that the RRT 0.34 and RRT 0.40 peaks represented the major degradation products of DWT and ETT. In order to identify the structure of the degradation products of RRT 0.34 and 0.40, we analyzed the pharmaceutical standards for the previously reported potent SOL degradation products (Solifenacin N-oxide, YM217880, isoquinoline, and isoquinoline ester) (Figure S2). The HPLC analysis revealed that the degradation products detected at RRT0.34 and 0.40 were solifenacin N-oxide and YM217880 (Figure S2), respectively, generated via oxidative reactions. Solifenacin N-oxide is produced via N-oxidation of the quinuclidine ring [19]. This is consistent with previous reports demonstrating that oxidation is the principal degradation mechanism of SOL. Approximately 20% of the drug is degraded when exposed to 5 M HCl solution, whereas approximately 80% of the drug is degraded when exposed to 3% hydrogen peroxide. These findings are also in line with previous reports showing that S1 (quinuclidin-8-yl-4-hydroxy-1-phenyl-3,4-dihydroisoquinoline-2(1H)-carboxylate, C23H27N2 O3), SII (2-(2-(phenyl((quinuclidine-8-yloxy)carbonylamino)methyl) phenyl) acetic acid, C23H27N2O4), and SIII (C23H27N2O3, N-oxide at the quinuclidine ring of SOL) were major degradation products of SOL under stress conditions [27]. In contrast, the DCT1 drastically improved the chemical stability of SOL compared to the DWT and ETT, with an identical composition. The total impurities (%) in the DWT and ETT after the four-week storage period were approximately 0.55 and 1.28%, respectively, whereas that of the DCT1 was calculated to be 0.17% (Figure 1B). There were also considerable differences in the formation of individual degradation products including RRT 0.34 and 0.40; the amount of degradation product with RRT 0.34 was estimated to be 0.22 and 0.23% for DWT and ETT, respectively, whereas its level in the DCT1 was determined to be 0.03% (Figure 1C). The levels of related substance with RRT 0.40 were also markedly higher in the DWT (0.11%) and ETT (0.45%) than in the DCT1 (0.015%) after four weeks of storage (Figure 1D). Therefore, we assumed that the use of solvents during the manufacturing process of the SOL-loaded tablets might promote drug degradation by enhancing the amorphization of the crystalline compound and subsequent oxidation and hydrolysis reactions. When solvents (ethanol or distilled water) are used during drug granulation, the succinate form of SOL is partially dissolved in the solvent and converted to a thermodynamically unstable amorphous state during the drying process. The formation of an amorphous form of SOL during a wet granulation-based fabrication process was reported; the amorphous form of SOL is more unstable than the crystalline form under high temperature and high humidity conditions [12]. Conversely, the DC technique reduces amorphization and the subsequent chemical reactions by eliminating drug dissolution and the subsequent conversion to an amorphous form during fabrication [29]. Based on these findings, we conclude that the DC technique could be an alternative to the wet granulation method for improving the stability of SOL-loaded tablets.

### 3.2. In Vitro Dissolution and Optimization of SOL-Loaded DCTs 

**Figure 2:**
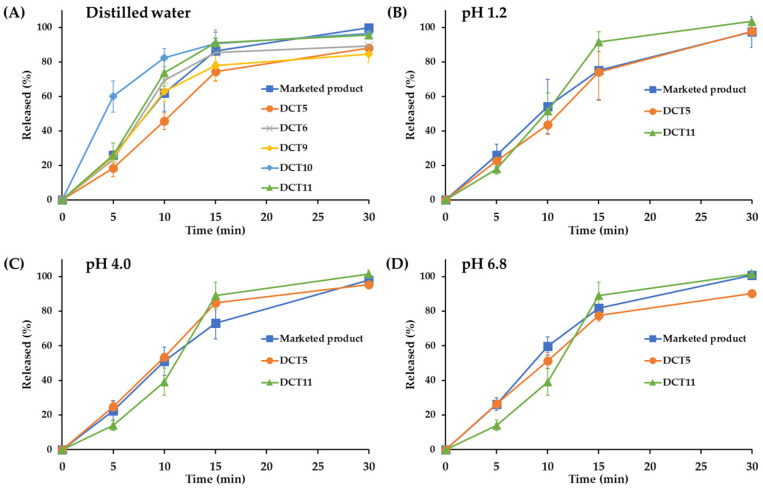
In vitro dissolution profiles of different SOL-loaded DCTs under (**A**) distilled water, (**B**) simulated gastric fluid (pH 1.2), (**C**) citrate buffer solution (pH 4.0), and (**D**) simulated intestinal fluid (pH 6.8). Note: data are represented as mean ± SD (*n* = 4).

After demonstrating that DC is an effective fabrication method for improving the chemical stability of SOL in a solid dosage form, a formulation study based on the DC technique was conducted to improve the drug stability of SOL while ensuring bioequivalence with the marketed product (Vesicare^®^, Astellas Pharma Inc., Tokyo, Japan) prepared using the wet granulation method. Although the DCT1 provided markedly improved storage stability compared to the DWT or ETT, the powder mixture had poor flow properties and was unsuitable for tabletization using a rotary tableting machine. In addition, picking and sticking were observed in the DCT1. To design SOL-loaded tablets based on the DC technique, different direct compressible, free-flowing diluents and disintegrants were used to evaluate the drug content, disintegration time, hardness, and friability of the tablets (DCT2–DCT5, Table 2). Lactose monohydrate, including Flowlac100 (spray-dried spherical lactose) and Supertab 30GR (fluid bed-processed agglomerate form), were combined with different grades of MCCs and disintegrants [30,31]. The particle size of the drug powder used in this study was determined to have a D_50_ of 80 μm, with D_10_ and D_90_ of 30 and 150 μm, respectively. In this study, different MCC and silicified MCC excipients were used, including Prosolv SMCC 90, Prosolv SMCC 50, Vivapur 12, and Vivapur 102. In addition, croscarmellose sodium, sodium starch glycolate, and Kollidone CL were screened as disintegrants. Kollidone VA64 is a common tablet binder and film-forming agent occasionally used as a sustained-release agent [32]. It was employed as a binder at 2.0 to 5.0% of the tablet weight. Sodium stearyl fumarate, a hydrophilic lubricant that is more SOL-compatible than magnesium stearate, was used in this study. The thickness and weight of the tablets were set to 3.1–3.3 mm and 154 mg, respectively, which were identical to those of the marketed product (approximately 154 mg). The hardness of the DCT2-DCT5 was determined to be 99.0–115.7 N, with tolerable mechanical strength (friability below 0.06%) (Table 2). Interestingly, there was a difference in the drug content depending on the type of lactose monohydrate; the DCT2 and DCT3, employing Flowlac100 as the diluent, showed a lower drug content than the Supertab 30GR-based tablets (DCT4 and DCT5); the drug content in the DCT2 and DCT3 were determined to be 92 and 86%, respectively, whereas those of the DCT4 and DCT5 were estimated to be 94.2 and 97.9%, respectively (Table 2). Flowlac100 is a spherical excipient prepared using spray-drying technology, whereas the drug powder possesses an irregular and angular surface (Figure S3). Thus, the excipient did not provide adequate interaction and intermixing with the drug powder, potentially causing static drug powder to be adsorbed and lost during the fabrication process. Conversely, Supertab 30GR, an agglomerated form of lactose monohydrate, retains a relatively rough surface with a particle size comparable to that of the drug powder [33]. Thus, the agglomerated form may offer uniform blending with the drug powder, impeding the adsorption and loss of the static drug powder during the fabrication process. In particular, when Supertab 30GR was combined with Prosolv SMCC 90 and Kollidone CL (DCT5), the drug content was higher than that of the DCT4, which contained Prosolv SMCC 50 and sodium starch glycolate. The in vitro dissolution profile of SOL in the DCT5 was evaluated using commercially available products. The drug dissolution profiles were evaluated using the paddle method (with a paddle speed of 50 rpm), which is widely used for the dissolution evaluation of immediate-release (IR) tablets [34]. Simulated gastric fluid (pH 1.2), buffer (pH 4.0), simulated intestinal fluid (pH 6.8), and distilled water were used as dissolution media. The commercial product provided rapid drug release; over 95% of the drug was released within 30 min at pH 1.2, 4.0, and 6.8, as well as in distilled water (Figure 2). The marketed product disintegrated within 10 min, and the water-soluble compound SOL rapidly dissolved in all the dissolution media, regardless of acidity. However, despite having a faster disintegration (7.3 min) than that of the marketed product, the drug release from the DCT5 was lower than that from the marketed product, with an extent of drug release of approximately 74 and 84% after 15 and 30 min, respectively (Figure 2). Therefore, additional DCT formulations were prepared to facilitate drug release from the tablets.

Different SOL-loaded tablets were formulated to accelerate drug release by adjusting the amount of binder (Kollidone VA64), lactose-to-MCC ratio, and the amount of disintegrant (Kollidone CL) (Table 2). Decreasing the amount of binder from 6 to 4 mg (DCT6) shortened the disintegration time of the SOL tablet from 7.3 to 5.7 min (Table 2). Accordingly, the drug release from the DCT6 tablet was greater than that from the DCT5, with 85 and 88% dissolution rates at 15 and 30 min in distilled water, respectively (Figure 2). However, the extent of the drug release after 30 min was much lower (>10%) than that of the marketed products. To further facilitate drug dissolution, the DCT7 (Supertab 30GR: Prosolv SMCC 90 = 5.5:1) and DCT8 (Supertab 30GR: Prosolv SMCC 90 = 23.3:1) had higher proportions of Supertab 30GR than the DCT5 (Supertab 30GR: Prosolv SMCC 90 = 2.2:1) (Table 2). Both the DCT7 and DCT8 provided appropriate mechanical properties (hardness between 108.9 and 109.8 N, friability between 0.05 and 0.16%) (Table 2). With an increase in the amount of hydrophilic excipient, the disintegration time of the DCTs drastically accelerated. The DCT7 and DCT8 showed disintegration times of 2.3 min and 1.7 min, respectively, which were more than 7 min faster than those of the marketed product (approximately 10 min), potentially exhibiting different drug dissolution and absorption rates in the gastrointestinal tract than those of the marketed product. 

As another approach to promote drug release, SOL-loaded tablets containing higher amounts of disintegrant (Kollidin CL, DCT9, and DCT10) or Aerosil 200 with Kollidon CL as a co-disintegrant (DCT11) were fabricated (Table 2). Increasing the disintegrant to 7.0 *w*/*w*% or using the combination of Aerosil 200 and Kollidon CL provided appropriate hardness (102.0–107.9 N) and friability (0.11–0.27%) (Table 2). The extent and rate of drug release from the DCT9 (5% Kollidon CLs) were not promoted; the amount of drug released after 15 and 30 min was approximately 78 and 84%, respectively, which was approximately 15% less than that of the marketed product (Figure 2). During an in vitro dissolution test of the DCTs, including the DCT5, DCT6, and DCT9, a coning phenomenon, that is, the formation of a mound of material at the bottom of the dissolution vessel, was observed, potentially retarding drug release from the solid dosage form. In contrast, when the amount of Kollidon CL increased to 7% in the DCT10, the disintegration of the tablet was drastically promoted to 2.6 min (Table 2). Subsequently, the release rate was markedly accelerated in all dissolution media, showing approximately 10–15% higher drug release than that of the commercial product after 15 min (Figure 2) with the removal of the coning phenomenon. To provide comparable disintegration and dissolution profiles between the DCT and the marketed product fabricated using the wet granulation method, a DCT containing Kollidon CL 3% along with Aerosil 200 1% was formulated (DCT11). The addition of colloidal silicone dioxide (Aerosil 200) resulted in a disintegration time of 6.7 min (Table 2), with a complete drug release after 30 min, regardless of the acidity of the dissolution media (Figure 2). A hydrophilic fumed excipient with a large surface area (200 m^2^/g) promoted tablet disintegration. The dissolution patterns of the DCT11 at pH 1.2, 4.0, and 6.8, and in distilled water were comparable to those of the marketed product, showing differences within 10% at all time points. The similarity between the dissolution profiles of the DCT11 and the marketed product was further evaluated by calculating the similarity factor (*f*_2_) [35]. Two dissolution profiles were considered similar when the *f_2_* value ranged from 50 to 100 [35]. The similarity factors (*f*_2_) between the dissolution profiles of the DCT11 and the marketed product at pH 1.2, 4.0, and 6.8, and in distilled water were calculated to be 53, 50, 65, and 61, respectively, indicating that the dissolution profile of the DCT11 was equivalent to that of the marketed product. Therefore, the DCT11 was chosen as the optimal DC composition for SOL, and a bioequivalence study with a commercial product was conducted.

### 3.3. Validation of LC/MS-MS Analysis Method of SOL in Human Plasma

**Figure 3:**
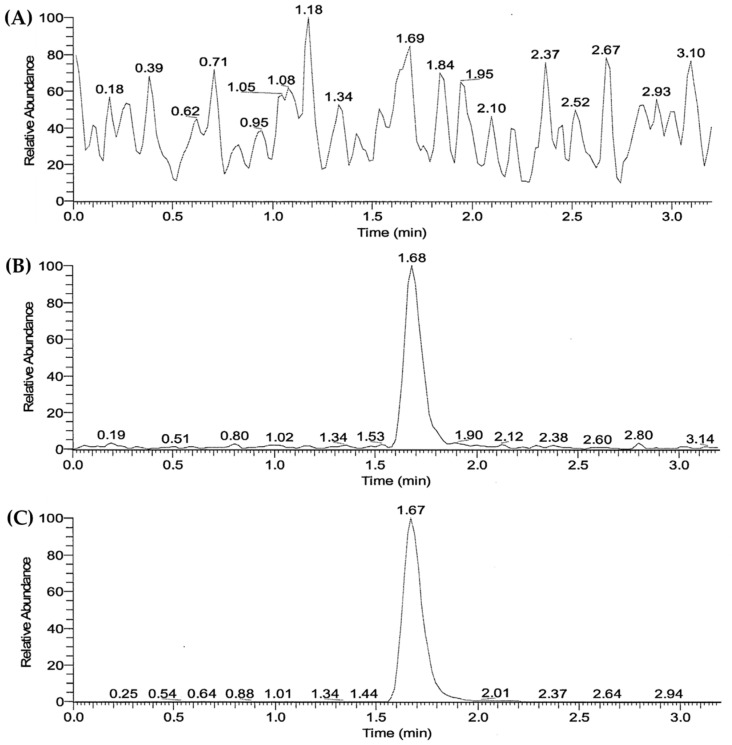
LC–MS/MS chromatograms of (**A**) blank plasma, and blank plasma spiked with (**B**) SOL and (**C**) IS, respectively.

The LC/MS-MS chromatograms of the plasma samples pretreated using the liquid–liquid extraction method are presented in Figure 3. The retention times of SOL and IS (SOL-d5) were determined to be 1.68 and 1.67 min, respectively. No interfering peaks were observed in the blank human plasma. The calibration curve obtained via linear regression analysis was linear (y = 0.117x − 0.000906691, *r*^2^ = 0.9990) in the drug concentration range of 1–50 ng/mL. The accuracy and precision of the established LC/MS-MS method were intra and inter-validated at the following four concentrations: low (0.2 and 0.6 ng/mL), medium (4 ng/mL), and high (40 ng/mL). As shown in Table 3, the intra- and inter-day accuracies of the four different concentrations ranged between 96.7 and 112.3%, and between 99.6 and 104.3%, respectively. Moreover, the intra- and inter-day precisions were determined to be 2.2–8.0% and 3.1–7.6%, respectively. These accuracy and precision results are within the acceptable criteria (accuracy ranging from 85 to 115% and precision within 15%) of the FDA guidelines [36]. Based on these findings, the established LC-MS/MS method was used to determine the plasma SOL concentration in a bioequivalence study.

### 3.4. Stability of the Optimized DCT under Accelerated Storage Condition

**Figure 4:**
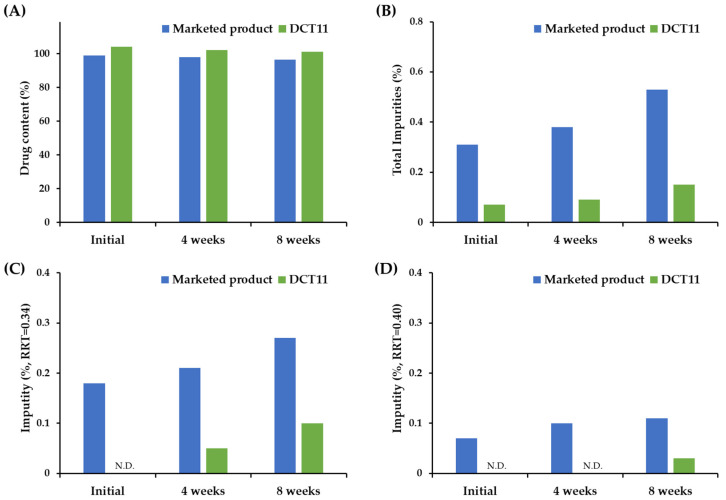
Comparison of chemical stabilities of SOL in the marketed product (Vesicare^®^) and the optimized DCT (DCT11) under accelerated storage condition (40 °C, RH75%). Changes in (**A**) drug content, (**B**) total impurities, individual impurity % at (**C**) RRT 0.34 and (**D**) RRT 0.40 in SOL-loaded tablets. Note: SOL-loaded tablets were packaged using alu-alu blister and stored in a stability chamber (40 °C, RH75%) for 4 or 8 weeks. To determine the drug content (%) and (**B**)-related substances (%) in the tablets, 10 tablets were pretreated simultaneously and analyzed. Impurities RRT 0.34 and 0.40 corresponded to solifenacin N-oxide and YM217880, respectively. N.D. = not detected.

The chemical stability of the DCT11, the optimal formula fabricated using the DC technique, and the marketed product under the accelerated storage condition (40 °C, RH75%) were further evaluated and are presented in Figure 4. Both formulations were stable, with no changes in the appearance, mechanical strength, or dissolution profile after 8 weeks of storage. Although the drug content in the tablets tended to decrease, it was maintained at > 95% for both tablets (Figure 4A). However, there was a marked difference in the levels of the degradation products formed between the DCT11 and the marketed product. For the marketed product, the initial total amount of related substances was determined to be 0.31% (Figure 4B), and the individual related substances (%) that peaked at RRT 0.34 and 0.40 were estimated to be 0.18 and 0.07%, respectively (Figure 4C,D). When stored under accelerated conditions, the total impurity (%) increased to 0.53% after 8 weeks, and the related substances (%) at RRT 0.34 and 0.40 increased to 0.27 and 0.11%, respectively. However, the DCT11 is chemically stable and markedly reduces the production of related substances during its fabrication and storage. The total impurities (%) after preparation and after 2 months of storage were 0.07 and 0.15%, respectively (Figure 4B). The level of individual degradation products at RRT 0.34 and 0.40 were also markedly decreased to 0.10 and 0.03%, respectively, after 8 weeks (Figure 4C,D). This indicates that the DC process can be a promising approach not only to simplify the manufacturing process, but also to improve the chemical stability of the drug during both the fabrication process and the storage period by preventing the surplus solubilization by the solvent; the transformation into the thermodynamically unstable amorphous form of the labile compound; and the potent chemical reaction with pharmaceutical excipients, oxygen, and moisture during storage.

### 3.5. Pharmacokinetics and Bioequivalence Study in Healthy Volunteers

**Figure 5:**
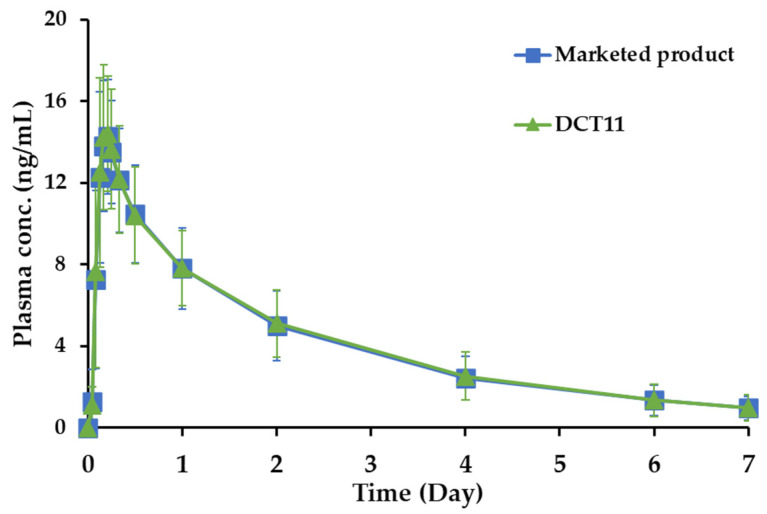
Plasma drug concentration–time profiles of SOL following oral administration (10 mg of SOL) of the marketed product (Vesicare^®^, ■) or DCT11 (▲) in healthy male participants. Data are represented as mean ± SD (*n* = 32).

The pharmacokinetic features of the DCT11 and the marketed product were compared in a randomized, single-dose, two-period crossover study in healthy male volunteers. The drug concentration–time profiles in the plasma following a single oral administration of DCT11 and the marketed product (10 mg SOL) are shown in Figure 5. The corresponding pharmacokinetic parameters, including C_max_, T_max_, AUC, and elimination T_1/2_, are presented in Table 4. Following the oral administration of both the SOL-loaded tablets, the drug concentration in the plasma increased gradually and reached a T_max_ between 3 and 6 h (T_max_ of DCT11 and Vesicare^®^, 4.3 and 4.5 h, respectively). The C_max_ values of the DCT11 and the marketed product were determined to be 15.3 and 14.9 ng/mL, respectively. Subsequently, the plasma SOL levels gradually decreased in both groups, and the drug concentrations were 7.8, 4.9, 5.1, 2.4, 2.5, and 1.3 ng/mL at 24, 48, 96, and 144 h after dosing, respectively. After 168 h, the plasma drug concentration was <1 ng/mL. The elimination half-lives (t_1/2_) of DCT11 and the marketed product were determined to be 48.0 and 49.4 h, respectively (Table 4). The T_max_ and t_1/2_ values obtained in our study coincided with the previous report indicating mean T_max_ and t_1/2_ values of 2.9 to 5.8 h (5 or 20 mg dosing) and 45.0 to 64.8 h, respectively, following the oral administration of the marketed product in healthy volunteers [37]. The AUC_0–196 h_ values, representing the systemic exposure of the drug and of the DCT11 and the marketed product, were measured to be 696.4 and 685.6 ng∙h/mL, respectively (Table 4). 

The bioequivalence of the DCT11 compared to the marketed product (10 mg) in healthy Korean male volunteers was further estimated by evaluating the relative ratio (T/R ratio) and 90% CI of the C_max_ and AUC_0–196 h_ values (Table 5). The T/R ratios for the log-transformed C_max_ and AUC_0–196 h_ of the DCT11, which were calculated by dividing the log-transformed C_max_ and AUC_0–196 h_ values of the marketed product, were 1.026 and 1.013, respectively (Table 5). The calculated 90% CIs of the geometric mean ratios of the test to the reference for the log-transformed C_max_ and AUC_0–196 h_ were 98.31–107.18% and 0.9761–1.0527%, respectively. The statistical data remained within the regulatory bioequivalence criteria of 0.8000–1.2500, indicating that the comparable in vitro dissolution profile offers an equivalent rate and extent of drug absorption despite the different inactive ingredients and fabrication processes [38]. Based on these findings, we conclude that the tablet formulated using the DC method is bioequivalent to the marketed product and can be prescribed as a chemically stable alternative to the marketed product.

## 4. Conclusions

A stable SOL-loaded tablet was fabricated using the DC technique with Supertab 30GR, Prosolv SMCC 90, Kollidone CL, Kollidone VA64, and Aerosil 200. The optimized DCT provided appropriate mechanical strength and an equivalent dissolution profile to the marketed product at pH 1.2, 4.0, 6.8, and in distilled water. In a pharmacokinetic evaluation of healthy male volunteers (a randomized, single-dose, two-period crossover design), the DCT and the marketed product exhibited comparable drug concentration–time profiles with statistical equivalence in the pharmacokinetic parameters (AUC and C_max_). Moreover, compared to the marketed product prepared via wet granulation technology, the tablets fabricated using the DC technique offer the advantage of a better drug chemical stability, releasing markedly lower levels of degradation products. Based on these findings, we conclude that the DC technique is a simple and effective method for formulating SOL-loaded tablets with improved storage stability.

## Figures and Tables

**Table 1:** Preliminary compositions and tablet features of SOL-loaded tablets prepared using different fabrication processes.

	DWT	ETT	DCT1
**Compositions (mg)**			
SOL	10.0	10.0	10.0
Lactose hydrate (200 mesh)	106.5	106.5	106.5
Corn starch	30.0	30.0	30.0
Hypromellose 2910	2.5	2.5	2.5
Distilled water	20	-	-
Ethanol	-	25	-
Magnesium stearate	1.0	1.0	1.0
Total (mg)	150	150	150
**Tablets features**			
Drug content (%) ^1^	99.4	100.3	98.2
Disintegration time (min) ^2^	7.8 ± 1.6	6.5 ± 2.1	3.5 ± 1.4
Hardness (N) ^3^	87.3 ± 2.2	78.5 ± 6.7	101.0 ± 8.5
Friability (%)	0.02	0.15	0.04

^1^ To determine the drug content (%) in the tablets, 10 tablets were pretreated and analyzed once. ^2^ Data represent mean ± SD (*n* = 6). ^3^ Data represent mean ± SD (*n* = 10). Notes: Sol-loaded tablets prepared using water- or ethanol-based wet granulation methods are denoted as DWT and ETT, respectively. The tablets prepared using the direct compression method are referred to as DCTs.

**Table 2:** Compositions and tablet features of SOL-loaded film-coated tablets prepared using DC technique.

	DCT 2	DCT 3	DCT 4	DCT 5	DCT 6	DCT 7	DCT 8	DCT 9	DCT 10	DCT 11
Compositions (mg)										
SOL	10.0	10.0	10.0	10.0	10.0	10.0	10.0	10.0	10.0	10.0
Flowlac100	89.5	89.5	-	-	-	-	-	-	-	-
Supertab 30GR	-	-	89.5	89.5	89.8	109.8	99.8	88.3	87.3	89.8
Vivapur-12	45.0	-	-	-	-	-	-	-	-	-
MCC pH102	-	40.5	-	-	-	-	-	-	-	-
Prosolv SMCC50	-	-	40.5	-	-	-	-	-	-	-
Prosolv SMCC 90	-	-	-	40.5	40.0	20.0	30.0	40.0	38.0	38.5
Sodium croscarmellose	-	4.5	-	-	-	-	-	-	-	-
Sodium starch glycolate	-	-	3.0	-	-	-	-	-	-	-
Kollidone CL	-	-	-	3.0	3.0	3.0	3.0	4.5	7.5	3.0
Aerosil 200	-	-	-	-	-	-	-	-	-	1.5
Kollidone VA64	6.0	6.0	6.0	6.0	4.5	4.5	4.5	4.5	4.5	4.5
Sodium stearyl fumarate	2.0	2.0	2.0	2.0	2.7	2.7	2.7	2.7	2.7	2.7
Opadry 03B640016	4.0	4.0	4.0	4.0	4.0	4.0	4.0	4.0	4.0	4.0
Total (mg)	-	154.0	154.0	154.0	154.0	154.0	154.0	154.0	154.0	154.0
Tablets features										
Drug content (%) ^1^	92.7 ± 1.3	86.7 ± 1.1	94.2 ± 1.0	97.9 ± 1.4	98.4 ± 1.4	97.4 ± 1.8	97.5 ± 1.0	99.6 ± 2.0	98.4 ± 0.7	100.1 ± 0.7
Disintegration (min) ^2^	12.1 ± 1.4	8.6 ± 0.8	8.4 ± 0.3	7.3 ± 1.1	5.7 ± 0.4	2.3 ± 0.3	1.7 ± 0.3	5.3 ± 0.6	2.6 ± 0.2	6.7 ± 0.8
Hardness (N) ^3^	103.0 ± 5.9	99.0 ± 4.9	109.8 ± 4.9	115.7 ± 5.9	98.1 ± 3.9	109.8 ± 3.9	108.9 ± 2.9	104.9 ± 6.9	102.0 ± 5.9	107.9 ± 9.8
Friability (%)	0.06	0.05	0.05	0.04	0.15	0.05	0.16	0.26	0.17	0.11

^1^ Data represent mean ± SD (*n* = 3). ^2^ Data represent mean ± SD (*n* = 6). ^3^ Data represent mean ± SD (*n* = 10).

**Table 3:** Precision and accuracy for inter- and intra-day LC–MS/MS analysis of SOL in human plasma.

			LLOQ (0.2 ng/mL)	Low (0.6 ng/mL)	Medium (4.0 ng/mL)	High (40 ng/mL)
Intra	1	Accuracy (%)	112.3	105.9	105.6	102.2
		Precision (C.V., %)	4.8	2.2	3.5	3
	2	Accuracy (%)	101.7	98.5	100.1	101.3
		Precision (C.V., %)	8	5.4	4.6	1.8
	3	Accuracy (%)	99.4	98.4	96.9	96.8
		Precision (C.V., %)	4.5	4.7	1.4	1.8
	4	Accuracy (%)	96.7	100.8	100	98
		Precision (C.V., %)	2.9	3.6	1.3	2.8
	5	Accuracy (%)	111.2	107.1	100.7	99.8
		Precision (C.V., %)	2.2	5.9	1.5	2.8
Inter	*n* = 25	Accuracy (%)	104.3	102.1	100.6	99.6
		Precision (C.V., %)	7.6	5.6	3.8	3.1

**Table 4:** Pharmacokinetic parameters of SOL in plasma following oral administration of the marketed product (Vesicare^®^) and the DCT11 in healthy male participants (10 mg of SOL, *n* = 32).

Parameters	Marketed Product	DCT11
C_max_ (ng/mL)	14.9 ± 2.9	15.3 ± 3.1
T_max_ (h)	4.6 ± 0.9	4.3 ± 1.0
AUC_0–196 h_ (ng·h/mL)	685.6 ± 214.2	696.5 ± 220.0
AUC_0–∞_ (ng·h/mL)	761.0 ± 267.1	772.7 ± 284.0
T1/2 (h)	49.5 ± 9.4	48.1 ± 11.2

Note: data represent arithmetic mean ± SD (*n* = 32).

**Table 5:** Statistical analysis of the bioequivalence between the marketed product (Vesicare^®^, 10 mg, ■) and the optimized DCT (DCT11, 10 mg, ▲) in healthy Korean male volunteers (*n* = 32).

Parameters	Marketed Product	DCT11	T/R Ratio	90% CI (log0.8–log1.25)
C_max_ (ng/mL)	1.166 ± 0.081	1.177 ± 0.088	1.01	log 0.98–log 1.07
AUC_0–196 h_ (ng·h/mL)	2.815 ± 0.139	2.822 ± 0.139	1.00	log 0.98–log 1.05

Note: data represent geometric mean ± SD (*n* = 32).

## Data Availability

Not applicable.

## Supplementary Materials

The following supporting information can be downloaded from https://www.mdpi.com/article/10.3390/pharmaceutics15061723/s1, Figure S1: HPLC chromatograms of the degradation product of solifenacin; Figure S2: Chemical structure of the degradation product of solifenacin; Figure S3: Optical microscopic images of the drug powder.
