# Supplementary material for: Novel Bioequivalent Tablet of Solifenacin Succinate Prepared Using Direct Compression Technique for Improved Chemical Stability
Source: Pharmaceutics. 2023 Jun 14;15(6):1723. doi: 10.3390/pharmaceutics15061723 (PMC10301382; doi:10.3390/pharmaceutics15061723)
Supplement: Supplementary file 1 [file pharmaceutics-15-01723-s001.zip › pharmaceutics-2391427-supplementary.pdf]

pharmaceutics

MDPI

Supplementary Materials: The following supporting information can be downloaded from: https://www.mdpi.com/article/10.3390/pharmaceutics15061723/s1.

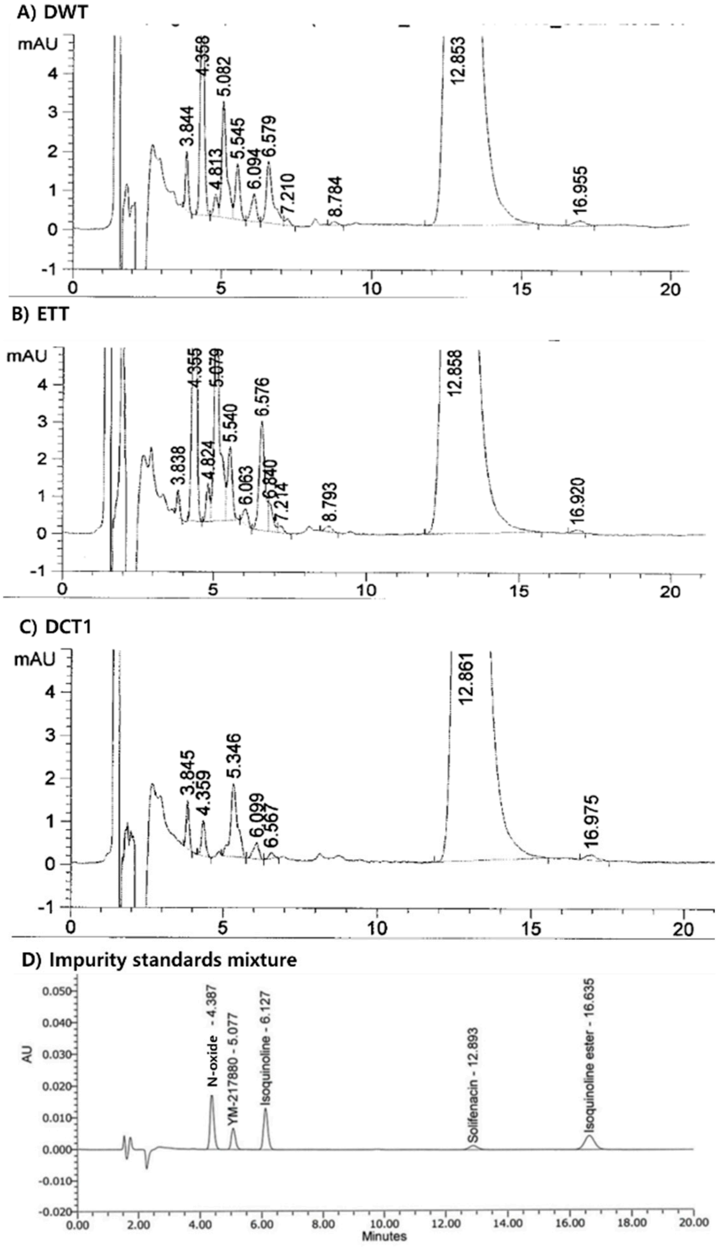

Figure S1. HPLC chromatograms of A) DWT, B) ETT, and DCT1 after two weeks of storage under accelerated conditions (40℃, RH75%), and D) pharmaceutical standard mixture of potent degradation products of SOL, such as solifenacin N-oxide, YM217880 ((+)-(R)-quinuclidin-3-yl [2-(2-benzoylphenyl)ethyl]carbamate), isoquinoline ((1S-1-Phenyl-1,2,3,4-tetrahydro-2-isoquinoline), and isoquinoline ester ((1S-ethyl-1-Phenyl-1,2,3,4-tetrahydro-2-isoquinoline carboxylate).

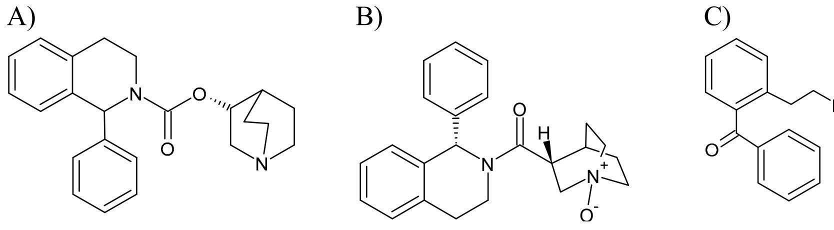

Figure S2. Chemical structures of A) SOL, B) solifenacin N-oxide, and C) YM217880 ((+)-(R)-quinuclidin-3-yl [2-(2-benzoylphenyl)ethyl]carbamate), the two major degradation products of the SOL-loaded tablets.

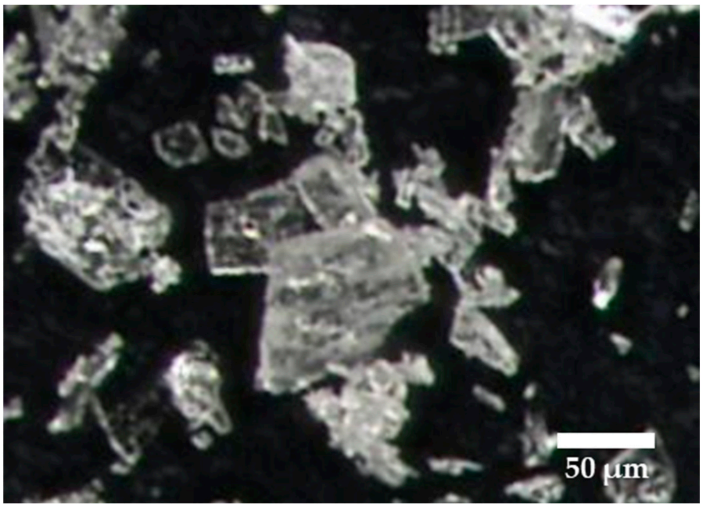

Figure S3. Optical microscopy images of the drug powder.
